# Commentary: Attentional control and the self: The Self-Attention Network (SAN)

**DOI:** 10.3389/fpsyg.2015.01726

**Published:** 2015-11-05

**Authors:** Adolfo M. García, David Huepe, David Martinez, Juan P. Morales, Daniela Huepe, Esteban Hurtado, Noelia Calvo, Agustín Ibáñez

**Affiliations:** ^1^Laboratory of Experimental Psychology and Neuroscience, Institute of Cognitive Neurology, Favaloro UniversityBuenos Aires, Argentina; ^2^National Scientific and Technical Research CouncilBuenos Aires, Argentina; ^3^Faculty of Elementary and Special Education, National University of CuyoMendoza, Argentina; ^4^UDP-INECO Foundation Core on Neuroscience, Diego Portales UniversitySantiago, Chile; ^5^Faculty of Psychology, National University of CórdobaCórdoba, Argentina; ^6^Universidad Autónoma del CaribeBarranquilla, Colombia; ^7^Centre of Excellence in Cognition and its Disorders, Australian Research CouncilSydney, NSW, Australia

**Keywords:** Self-Attention Network model, own-name effect, own-face effect, familiarity, ventromedial prefrontal cortex (vmPFC)

The Self-Attention Network (SAN) model (Humphreys and Sui, 2015) is a recent neurocognitive model to account for self-biases in the allocation of attention. It emerges from psychological, neuropsychological, and neuroimaging evidence on three phenomena: own-name effects, own-face effects, and self-biases in associative matching. Specifically, it posits that our responses to self-related stimuli are differentially subserved by a network comprising three nodes: (i) a general-purpose top-down attentional control network which involves the dorsolateral prefrontal cortex and the intra-parietal sulcus; (ii) a self-representation hub located in the ventromedial prefrontal cortex (vmPFC), and (iii) a bottom-up orientating mechanism which depends on the posterior superior temporal sulcus (pSTS). Accordingly, attentional shifts upon hearing our own name or seeing our own face would rely on interactions among such nodes, mimicking perceptual-saliency effects, and determining emergent behavior.

Though attractive, this proposal features two major caveats. First, the evidence for own-name effects is inconsistent and undermined by psycholinguistic confounds. Second, the node proposed to subserve self-specific information lacks neurofunctional specificity. Here we discuss both issues and advance relevant methodological recommendations.

The model resorts to own-name studies allegedly showing biases for self-related information. However, confirmatory evidence has not been consistently replicated (Yang et al., 2013), especially when own names are compared to other familiar names (e.g., Harris et al., 2004; Kawahara and Yamada, 2004; Tacikowski et al., 2011). Moreover, when they do emerge, own-name effects may be related to non-self-specific psycholinguistic confounds, such as familiarity and frequency. Familiar and frequent words are processed faster (Guttentag and Carroll, 1994) and yield distinctive electrophysiological modulations (Kutas and Federmeier, 2011). Subjectively, own names are typically more frequent and familiar than other proper and common names. Thus, it is difficult to rule out the impact of such variables on the observed effects.

Moreover, there is no neuroanatomical evidence for a dissociation between own names and other proper or familiar names. A review of patients with selective deficits in recalling people's names found no support for a specialized mechanism supporting proper-name—let alone own-name—processing (Hanley and Kay, 1998). Finally, neuroimaging and lesion studies indicate that proper-name processing mainly involves left temporal structures and, less notably, subcortical structures (Semenza, 2006). None of these regions is part of the neuroanatomical components of the SAN model, and there is no rationale for why one's own name should rely on a network separate from that specialized in processing its overarching category. At the very least, the model should specify the relationship between the regions proposed to support own-name biases and the broader networks subserving lexical processing, in general, and proper names, in particular.

The SAN model further posits that self-specific information is subserved by a putative brain region. The model includes “a self-representation hub *housed* in the ventromedial prefrontal cortex (vmPFC)” (Humphreys and Sui, 2015: 15; emphasis ours). However, this association lacks neurofunctional specificity. The vmPFC is critically engaged by *any* affective response shaped by conceptual information about specific outcomes (Roy et al., 2012), and by bottom-up processing of external and internal salient stimuli (Cona et al., 2015). Crucially, the vmPFC is key to assess the familiarity of others' faces (Gilboa et al., 2009) and to discriminate them as a function of their relevance (Pegors et al., 2015). Finally, when one's own name is compared with stimuli of similar personal relevance (e.g., the name of a significant other), vmPFC activity is not differentially modulated by self-biased information. So, rather than self-attention in particular, vmPFC activations seem to index increased affective meaning, relevance, or familiarity of self-faces. In brief, the vmPFC does not seem specific enough to constitute a distinct node subserving *sui generis* self-attention.

A second caveat concerning the role of the vmPFC has been noted by Vallesi (2015). As this author argues, the model assumes strong and mostly unidirectional excitatory connections from the vmPFC to the pSTS. However, this claim is incompatible with lesion data showing that self-bias effects decrease after damage to the former region, but increase following damage to the latter (Sui et al., 2015a). Accordingly, Vallesi (2015) posits that vmPFC activity may be modulated via inhibitory feedback connections from the pSTS, a specification that is not captured by the putative SAN model.

These caveats may be circumvented via methodological innovations. The confounds surrounding own-name research may be avoided through “new nickname” studies. Participants could be given *ad hoc* nicknames and be referred to by them systematically throughout an experimental session. All names would have the same frequency and familiarity at baseline (namely, zero), and they could be matched for other relevant psycholinguistic variables, such as length or phonological complexity. If an advantage for own nicknames is thus observed, claims for a self-attention bias could be more validly entertained. A conceptually similar paradigm, designed by the very proponents of the SAN model (Sui et al., 2012, 2015b), illustrates the potential usefulness of “new nickname” studies. For example, upon establishing arbitrary associations between geometric shapes and themselves or other people, participants then show reliable self-prioritization effects, independent of psycholinguistic confounds (Sui et al., 2012). This evidence supports the possible benefits of “new-nickname” studies: while these would involve a similar design, they would decrease perceptual-matching demands and more directly address biases in the specific domain of proper-name processing.

Regarding neuroanatomical concerns, functional and structural connectivity analyses would help clarify whether there is a relationship among SAN hubs and regions for selective processing of own names/faces. Moreover, the (relative) specificity for self-information in the vmPFC could be tested by comparing stimuli with similar relevance and familiarity but with different degrees of self-related information. Finally, additional neuropsychological as well as effective and functional connectivity studies could help elucidate the role of excitatory and inhibitory connections between the vmPFC and the pSTS, showing whether the latter structure modulates self-information processing in the former (Vallesi, 2015). Until these limitations are addressed, the SAN model will remain psycholinguistically and neuroanatomically underspecified.

## Conflict of interest statement

The authors declare that the research was conducted in the absence of any commercial or financial relationships that could be construed as a potential conflict of interest.
